# Systems-Level Smoking Cessation Activities by Private Health Plans

**Published:** 2010-12-15

**Authors:** Sharon Reif, Constance M. Horgan, Deborah W. Garnick, Deborah L. McLellan

**Affiliations:** Institute for Behavioral Health, Heller School for Social Policy and Management, Brandeis University; Brandeis University, Waltham, Massachusetts; Brandeis University, Waltham, Massachusetts; Brandeis University, Waltham, Massachusetts

## Abstract

**Introduction:**

The US Public Health Service urges providers to screen patients for smoking and advise smokers to quit. Yet, these practices are not widely implemented in clinical practice. This study provides national estimates of systems-level strategies used by private health insurance plans to influence provider delivery of smoking cessation activities.

**Methods:**

Data are from a nationally representative survey of health plans for benefit year 2003, across product types offered by insurers, including health maintenance organizations (HMOs), preferred provider organizations, and point-of-service products, regarding alcohol, tobacco, drug, and mental health services. Executive directors of 368 health plans responded to the administrative module (83% response rate). Medical directors of 347 of those health plans, representing 771 products, completed the clinical module in which health plan respondents were asked about screening for smoking, guideline distribution, and incentives for guideline adherence.

**Results:**

Only 9% of products require, and 12% verify, that primary care providers (PCPs) screen for smoking. HMOs are more likely than other product types to require screening. Only 17% of products distribute smoking cessation guidelines to PCPs, and HMOs are more likely to do this. Feedback to PCPs was most frequently used to encourage guideline adherence; financial incentives were rarely used. Furthermore, health plans that did require screening often conducted other cessation activities.

**Conclusion:**

Few private health plans have adopted techniques to encourage the use of smoking cessation activities by their providers. Increasing health plan involvement is necessary to reduce tobacco use and concomitant disease in the United States.

## Introduction

Tobacco use is a leading preventable cause of death and extensive health-related economic losses (1). Tobacco use cessation efforts are highlighted by the 2008 US Public Health Service smoking cessation clinical practice guideline (2). Effective treatments exist, and providers influence smoking cessation rates when they encourage their patients to quit smoking (2), which is recommended for quality care delivery (3). Yet, a large gap is found between the existence of guidelines and recommendations and their implementation in clinical practice (4,5).

The clinical practice guideline emphasizes the need for systems-level approaches that can increase provider intervention and reduce smoking (2,6). It calls on administrators, insurers, and purchasers to provide leadership in implementing systems interventions that include provider training, resources, and feedback. It also recommends insurance coverage and physician reimbursement for tobacco dependence treatment (2). Such systems interventions are necessary for preventive medicine and chronic disease management (7).

Health plans, which offer specific products or packages of defined benefits to their purchasers (8,9), may be influenced to adopt systems-level strategies. Purchaser demand often drives what is covered by those products, and purchasers can ensure specific activities by requiring them in the contract, although they usually do not demand smoking cessation services (10,11). Additionally, screening activities can be considered a basic indicator of the quality of the health plan, as indicated by inclusion of measures of medical assistance with smoking cessation — advising smokers to quit, discussing medications, and discussing strategies — in the National Committee for Quality Assurance's Healthcare Effectiveness Data and Information Set (HEDIS) accreditation measures (12,13). The HEDIS measures may be an incentive to health plans to offer screening, cessation advice, and referral.

Health plans, in turn, may influence the behaviors of providers and enrollees. About 60% of Americans have employer-based health insurance, largely through managed care organizations (14,15). Twenty percent of privately insured adults are current smokers (16). Thus, private health plans have an important role for adoption and implementation of clinical practice guidelines. By implementing systems-level changes, health plans can influence providers to help their patients quit smoking. In addition, health plans have been shown to independently influence HEDIS quality measures, beyond actions of providers, for patients across multiple health maintenance organizations and provider groups (17).

We used a nationally representative survey of health plans to examine systems-level strategies for smoking cessation, considering screening and guideline distribution practices in particular, across a full product range offered by private health plans (health maintenance organizations [HMOs], point-of-service [POS] products, and preferred provider organizations [PPOs]). Prior studies have examined provider activities or patient knowledge or receipt of services, excluded some types of health plans, or were not nationally representative (4,6,7,10,18-22). We investigated how health plans are implementing systems-level approaches to smoking cessation.

## Methods

### Data source and sample

We conducted a nationally representative telephone survey of 368 private health insurance companies in 60 market areas for benefit year 2003 (23). The aim of the overall study was to understand how private health plans provide alcohol, drug, and mental health services, and how this has changed over time. These data, collected periodically by our team, are the most recent nationally representative data available about alcohol, drug, and mental health services in private health plans. The Brandeis University institutional review board approved the study.

The study used the sampling frame from the Community Tracking Study, a longitudinal study of health system changes and their effects on people (24). The primary sampling units for this study were the 60 Community Tracking Study market areas selected to be nationally representative; health plans were then selected within market areas. Plans serving multiple markets were defined as separate health plans for the study to ensure that a market area was fully characterized; data were collected with reference to the specific market area. At each health plan, we asked respondents about the top 3 products they offered, defined as "packages, plans, or contracts that are similar in terms of out-of-network coverage, referrals, and primary care physicians." For each product, we asked respondents about the "most commonly purchased package."

To be eligible, a health plan had to offer a managed care product in the market area, have at least 300 subscribers or 600 covered lives in the market area, not function exclusively as a third-party administrator, and have at least 1 eligible product. Eligibility for a product within the health plan included having at least 100 subscribers or 200 covered lives in the market area, not enrolling exclusively Medicaid or Medicare patients, being sold directly to purchasers such as employers, not being exclusively a third-party administrator product for the health plan, being a managed care product or consumer-driven health plan, and offering coverage for a full range of health and behavioral health services.

The sample totaled 814 plans, of which 441 were deemed eligible. Ineligible plans had closed, merged, or were otherwise unreachable (n = 146); did not offer comprehensive health care products (n = 106); had low enrollment (n = 76); or were ineligible for several other reasons (n = 45). Of eligible plans, 368 (83%) responded to the administrative module, reporting on 812 products. Of responding plans, 347 (79% of eligible health plans, and 94% of health plans completing the administrative module) completed the clinical module, reporting on 771 products. Nonrespondents were more likely to offer HMO products. Four consumer-driven plans, which allow enrollees to design their own network and benefit options, were excluded from analyses because they represent a very different approach than the other product types, and few existed.

The survey focused on alcohol, drug, and mental health services; several questions were about smoking cessation in primary care settings. It had an administrative module addressing behavioral health contracting, benefits, network management, and provider payment, and a clinical module addressing primary care screening and treatment, specialty treatment, utilization management, prescription drugs, and quality improvement. The administrative and clinical modules were conducted sequentially, took about 45 minutes each to complete, and collected detailed product-level data for each topic. Typically the health plan's executive director responded to the administrative module and referred us to the medical director to complete the clinical module. In some cases, the health plan requested that the managed behavioral health organization, with which plans sometimes contracted to provide alcohol, drug, and mental health services, provide information for the clinical module. Data were collected from April 2003 through April 2004.

Health plan products were classified by product type: HMOs, in which services are provided by a network of affiliated providers, and services outside the network are generally not covered; POS products, in which both in-network or out-of-network services may be chosen, albeit with different coinsurance payments or deductibles; or PPOs, in which enrollees are given a financial incentive, usually a different coinsurance payment or deductible, to use a preferred network of providers.

### Measures

The clinical module, typically answered by the health plan’s (or managed behavioral health organization’s) medical director, included a series of questions related to smoking, which allowed consideration of select systems-level smoking activities as implemented by the health plan. The first set of questions focused on screening for smoking: 1) Were primary care providers (PCPs, which include physicians, nurses, and other physician extenders) required by the health plan to screen for smoking among at least some of their patients? 2) Were PCPs required by the health plan to use a general health screening questionnaire that included questions about smoking? and 3) Did the health plan verify that screening for smoking was done by PCPs, and if so, was there a system to report the results of that screening? The second set of questions focused on written guidelines for smoking: Did health plans distribute written guidelines specifically for primary care treatment of smoking? If so, which techniques were used by the health plan to encourage PCPs to adhere to the guidelines: financial incentives, training about guidelines, feedback regarding their own performance relative to guidelines, or feedback about guideline adherence by network providers overall? Specific survey items used in these analyses may be found in the Appendix.

### Statistical analysis

We analyzed data with SUDAAN version 9.0 (RTI International, Research Triangle Park, North Carolina) for sampling variance estimation, given the complex sampling design. The reported findings are national estimates, weighted to be representative of health plans' private managed care products in the continental United States. The sampling weights applied to produce national estimates were computed from the inverse of the selection probabilities, which were computed from each stage of selection: site selection (exactly the probability used in the Community Tracking Study) and the selection of the plans in each site. Nonresponse adjustment and iterative proportional fitting were used to calculate the final weights. We used pairwise *t* tests to determine significance of screening behaviors for each product type (Table) and whether screening was required; health plans with missing data were excluded only from the specific analysis in which those data were missing (ie, exclusions did not occur listwise).

### Item response rate

The item response to several smoking questions was lower than for other questions in the clinical module. Smoking is often considered a general medical rather than a behavioral health concern, so some respondents to this survey about behavioral health services may not have known about more general smoking-related activities in the health plan. This situation would be most likely when the managed behavioral health organization was asked to respond.

## Results

Health plans were split fairly evenly among product types; slightly fewer identified their products as HMOs (29%) than POS products (36%) or PPOs (35%). Nearly three-fourths of products were in health plans that contracted with a managed behavioral health organization for their alcohol, drug, and mental health services. Most (86%) plans were for-profit.

### Screening for smoking

Few products require screening for smoking in primary care settings (Table). Only 9% require that PCPs screen for smoking among at least some clients. Slightly more products require the use of a general health screening questionnaire that includes smoking (11%) or verify that PCPs screen for smoking (12%). If they do verify screening, most have a system in place to report those results.

While screening activities for smoking are low overall, HMOs are more likely than either POS or PPO products to require screening among at least some patients (15%), require screening via a general questionnaire (17%), and verify that screening is conducted (19%). However, if they verify that screening is conducted, HMOs are less likely than other products to have a system in place to report the results.

### Distribution of written smoking cessation guidelines

More products report distributing guidelines than requiring screening, although the proportion is low (Table). Overall, only 17% of products distribute smoking cessation guidelines specific to primary care. As with screening, HMOs are more likely to do this; 24% distribute guidelines, compared with 12% of POS products and 16% of PPO products.

If guidelines are distributed, a variety of techniques may be used to encourage adherence to them (Table). Few products offer financial incentives to PCPs. Nearly one-third of products overall offer training on guidelines, and only one-fifth of PPO products do. The most frequently used tool to encourage adherence to guidelines was feedback to PCPs. Of products that distributed written guidelines, 37% provided feedback regarding the provider's own performance and more than half (53%) provided feedback about overall network provider adherence to guidelines.

### Multiple screening activities

Products that require screening for smoking among at least some patients are also significantly more likely than those that do not require screening to conduct other screening and distribution activities. Among those that require screening, 51% to 62% also require screening in a general health questionnaire, verify that screening is done, or distribute written guidelines, while less than 11% of those that do not require screening conduct these same activities. Thus, participation in 1 activity is associated with increased participation in the others.

## Discussion

Systems-level support for smoking cessation is not widespread among private health plans. Despite current clinical guidelines and recommendations, most plans do not require providers to screen for smoking, determine whether screening is done, or distribute relevant guidelines to providers.

Some studies based on enrollee reports have found high rates of identifying smokers (10,13,21), but our results based on health plans are consistent with a 2001 survey of physician organizations, which found few physicians are required to provide smoking cessation activities, and very few receive financial incentives from health plans (25). America's Health Insurance Plans reports that two-thirds of health plans have written guidelines for smoking cessation (10), but we found that few actually distribute them. The positive news from our study is that health plans that make an effort to screen for smoking report multiple smoking cessation activities, an indication of a multipronged approach to improve smoking cessation rates. Such a systemic approach is likely to improve provider behavior.

When techniques were used to encourage adherence to guidelines, we found that plans most often compared results among providers, but financial incentives were rarely used; this was similar to other findings (10). In other research, both provider feedback and financial incentives were related to significantly more smoking cessation activities reported by patients, and greater knowledge of and assistance in these activities by providers (25-27). Health plans that increase use of these techniques may improve their enrollees' smoking rates. However, even when health plans use some of these techniques, providers may be unaware of them, indicating room to improve adoption and implementation (28). Health plans could also step beyond the provider to offer, for example, incentives directly to enrollees, online smoking cessation programs, or programs within a broader wellness approach.

Why are health plans not doing more in this arena, when it has been shown that health plan strategies can independently affect activities such as screening (17)? Lack of provider compliance is cited as a barrier to increased activities by health plans (10). We found that slightly more health plans verify screening than require screening, perhaps in response to this perceived barrier of poor compliance. Moreover, a health plan requirement may be considered a burden for providers (14) or not realistically enforceable. Health plans also report specific systems-level barriers to addressing smoking cessation, including competing priorities, issues with data and reporting systems, and lack of resources, staffing, or funding (10,29). Health plans may assume that responsibility lies elsewhere, such as with providers or the public health system, despite the call for systems-level approaches that include insurers (2). Purchasers largely do not demand smoking cessation interventions as part of their health care packages (10,11), yet purchaser demand can drive health plan activities. Health plans may doubt the business case for smoking cessation activities: 61% of plans report delayed return on investment as a barrier (10). Yet, the literature suggests that tobacco use cessation activities are cost-effective for both health plans and employers and provide a reasonable return on investment (2,30,31).

Despite overall low rates of required screening for smoking and distribution of written guidelines, HMOs were significantly more likely than other products to report each activity. The original HMO mission often highlighted prevention as well as treatment (32,33); thus, smoking cessation activities may fit better there than in other types of health plans. It is also perhaps easier to conduct these activities within the constraints of the more managed provider network used by most HMOs. That is, HMO providers might be more aware of the health plan rules and guidelines compared with providers in a loosely networked PPO who participate in multiple health plans. HMOs may also be more likely to emphasize smoking cessation activities because they are measured as part of HEDIS (12) and thus may be viewed as valuable to accreditation; HEDIS did not apply to PPOs at the time of the survey. In 2003, HEDIS showed that 69% of smokers who saw a physician were advised to quit, yet only 36% discussed specific strategies with their physician (13). Although not directly comparable to our findings of health plan activities, because they are ascertained via enrollee surveys and do not identify the systemic approaches that health plans may use, HEDIS measures may improve with a systems focus on smoking cessation by health plans.

We note several potential limitations. First, this survey focused on alcohol, drug, and mental health services and may have been completed by someone more familiar with these services and less familiar with general medical services, such as a representative of a separately contracted managed behavioral health organization. If smoking cessation is in the purview of general medical services (ie, those provided by PCPs) then some respondents may not have been aware of those activities, as indicated by missing data, and we have potentially underestimated the prevalence of cessation activities. However, even if all missing data had been a positive response, the prevalence of smoking cessation activities by health plans would still be low. Second, this is a survey of health plans, not providers, so it is unknown how successfully providers in these plans are screening their patients and how this varies by health plan requirements. Third, although these data from 2003 are still the most recently available about health plans, changes over time may have occurred. Fourth, screening is only the first step in a range of recommendations that lead to smoking cessation.

Even if all health plans required screening, verified that their providers did it, and distributed treatment guidelines, health plans should encourage and monitor smoking cessation activities and follow-up (29), moving beyond calls for improved delivery of services by providers (34,35). Such activities could include requiring that providers not only ask their patients whether they smoke but also advise them to quit and then provide assistance with quitting through either medication or quitline services. Health plans should pay for smoking cessation services, which is an incentive to providers to offer them. Billing codes specific to tobacco cessation counseling were added to the Healthcare Common Procedure Coding System in 2005 and Current Procedural Terminology in 2008. The addition of the codes to these standard billing code systems used by insurers and providers allows providers to be paid for smoking cessation services and gives plans a way to indicate their coverage of these services. However, it is unclear how many health plans accept these codes for reimbursement. Unless the codes are adopted across plans, providers, who largely accept multiple types of insurance and may not know the insurer for any given patient (34), may be unlikely to use the codes and to offer smoking cessation services for which they are not reimbursed.

These data from the 2003 benefit year, the most recently available national data on private health plans, are still relevant to consider how systems-level interventions can affect providers' ability to change behavior in their patients. Our findings of limited systems-level efforts by health plans to promote smoking cessation activities by their providers suggest several conclusions. First, systems approaches to smoking cessation still need to be enhanced and adopted (2,18,36). Second, purchasers and participants need to demand systems-level smoking cessation activities (35). Third, research should investigate effective incentives and techniques that health plans could use to change behavior among providers and enrollees. Fourth, a variety of smoking cessation strategies, as highlighted by Orleans (35), should continue to be encouraged. Future research should consider how health plan activities interact with activities of other systems, such as the public health system, and of providers themselves. It should also address health plans' concern with return on investment as a barrier to these activities (34). In the meantime, by encouraging health plans to focus on smoking cessation at the systems level, further inroads can be made to reduce the burden of smoking on enrollees, employers, and health plans themselves.

## Figures and Tables

**Table:** Screening Activities for Smoking and Written Guidelines for Smoking Cessation in a National Survey of Private Health Plans, 2003^a^

Screening Activities	Product Type, No. (%)				*P* Value^b^		

	All, n = 767, Weighted n = 7,530	HMO, n = 247, Weighted n = 2,209	POS, n = 261, Weighted n = 2,702	PPO, n = 259, Weighted n = 2,619	HMO vs POS	HMO vs PPO	POS vs PPO
**PCPs required to screen for smoking among some patients^c^**	632 (9)	308 (15)	177 (7)	147 (6)	.003	<.001	.75
**PCPs required to use general health screening questionnaires that include smoking^d^**	684 (11)	306 (17)	187 (8)	191 (9)	.003	.001	.71
**Verify that PCPs screen for smoking^e^**	816 (12)	392 (19)	278 (11)	146 (7)	.009	<.001	.42
**Have system to report results of verification**	696 (85)	318 (81)	241 (86)	137 (94)	.31	.02	.32
**Written smoking cessation guidelines for primary care treatment are distributed^f^**	1,009 (17)	432 (24)	278 (12)	298 (16)	<.001	.002	.08
**If guidelines are distributed, the following are given to PCPs to encourage their use:**							
Financial incentives^g^	60 (6)	7 (2)	53 (19)	0	.29	.23	.24
Training about guidelines^h^	295 (31)	150 (37)	85 (31)	60 (22)	.48	.02	.26
Feedback about PCPs' performance relative to guidelines^i^	294 (37)	114 (35)	124 (46)	56 (28)	.45	.50	.18
Feedback about general network provider adherence to guidelines^j^	411 (53)	175 (56)	164 (61)	72 (39)	.64	.07	.06

Abbreviations: HMO, health maintenance organization; POS, point-of-service product; PPO, preferred provider organization; PCP, primary care provider.

a Products are defined as packages, plans, or contracts that are similar in terms of out-of-network coverage, referrals, and PCPs within a given health plan. Reported percentages exclude products for which data were missing; all data are weighted.

b Calculated by using pairwise *t* tests.

c Missing 5% (n = 372) of products (HMO n = 127, POS n = 83, PPO n =162).

d Missing 17% (n = 1,292) of products (HMO n = 409, POS n = 370, PPO n = 513).

e Missing 9% (n = 690) of products (HMO n = 129, POS n = 94, PPO n = 467).

f Missing 20% (n = 1,506) of products (HMO n = 371, POS n = 353, PPO n = 782).

g Missing 1% (n = 6) of products (HMO n = 6, POS n = 0, PPO n = 0).

h Missing 5% (n = 49) of products (HMO n = 22 , POS n = 6, PPO n = 21).

i Missing 20% (n = 211) of products (HMO n = 106, POS n = 6, PPO n = 99).

j Missing 24% (n = 237) of products (HMO n = 117, POS n = 6, PPO n = 114).

## Appendix. Selected Survey Questions Regarding Systems-Level Smoking Cessation Activities

1.4 And what type of product would you say [PRODUCT NAME] is? Is it an HMO, PPO, POS, indemnity product, a consumer-driven plan or is it something else?

**PROBE:** Please select the product type that ***most closely*** resembles [PRODUCT] in terms of out-of-network coverage and referrals. **IF NEEDED, READ DEFINITIONS.**

7.1 First I'm going to ask about screening and treatment for mental health, alcohol and drug problems, and smoking in [SITE].

Under [PRODUCT], do you **check** to see if screening is done by primary care practitioners for any of the following?

**PROBE:** "Screening" is defined as the identification of a problem in patients who are not yet known to have it.
**PROBE:** If you check whether screening is done, but only for a subset of providers, eg, high-volume providers, please indicate "Yes, some PCPs."
**PROBE:** Primary care practitioners can include physicians as well as nurses or other physician extenders.

- Mental health problems such as major depression or anxiety disorder
- Alcohol problems
- Drug abuse problems
- Smoking

7.4 Now we are going to ask about screening that the health plan requires in primary care settings. We begin with questions about general health screening and then turn to screening for specific mental health problems, alcohol and drug abuse, or smoking.

Under [PRODUCT], are primary care practitioners **required** to use **general health** screening questionnaires that include questions about any of the following?

**PROBE:** By "required," we mean that the health plan conveys the expectation that an action will be performed. Requirements that are not enforced may still be considered requirements.

- Mental health problems
- Alcohol problems
- Drug problems
- Smoking

7.11 Under [PRODUCT], are primary care practitioners **required** to screen for **smoking** among at least some of their patients?

7.15 Now I have some questions about the treatment of mental health, alcohol and drug problems, and smoking by primary care providers*.*

Under [PRODUCT] in [SITE], is there distribution of written guidelines specifically for primary care treatment of any of the following problems?

**PROBE:** By distribute, we mean electronic transmission or paper mailings.
**PROBE:** Guidelines are defined as standards used to guide providers based on accepted clinical treatment protocols for typical cases.

- Depression (MH)
- Anxiety (MH)
- Eating disorders (MH)
- Alcohol or drug abuse problems (SA)
- Smoking cessation (SM)

7.18 **[SKIP if 7.15 = no]** For [PRODUCT], which, if any, of the following are given to primary care providers to encourage their adherence to these guidelines?

**INTERVIEWER: ONLY ASK ABOUT GUIDELINES THAT ARE DISTRIBUTED FOR MENTAL HEALTH (MH), ALCOHOL OR DRUG ABUSE TREATMENT (SA), OR SMOKING CESSATION (SM) — ANY IN Q7.15 IS YES, OTHERWISE SKIP TO NEXT COLUMN.**

- Financial incentives connected to guideline adherence
- Training about guidelines
- Feedback regarding their own performance relative to guidelines
- Feedback about guideline adherence by network providers in general
